# Nam Dia long, a Vietnamese folk formula, induces apoptosis in MCF-7 cells through various mechanisms of action

**DOI:** 10.1186/s12906-017-2027-2

**Published:** 2017-12-04

**Authors:** My-Nuong Thi Nguyen, Thuy-Duong Ho-Huynh

**Affiliations:** 1 Department of Genetics, Faculty of Biology and Biotechnology, University of Science, VNU-HCM, 227 Nguyen Van Cu Street, Ward 4, District 5, Ho Chi Minh City, Vietnam; 2 Molecular Genetics and Genomics Research Group, Cancer Research Laboratory, University of Science, VNU-HCM, Building B6.1, Dong Hoa Village, Di An District, Binh Duong Province, Vietnam

**Keywords:** Traditional medicine, Breast cancer, Apoptosis, Microarray profiling, Nam Dia long

## Abstract

**Background:**

The holistic approach of traditional medicine renders the identification of its mechanisms of action difficult. Microarray technology provides an efficient way to analyze the complex genome-wide gene expression of cells treated with mixtures of medicinal ingredients. We performed transcriptional profiling of MCF-7 cells treated with Nam Dia Long (NDL), a Vietnamese traditional formula, to explore the mechanism of action underlying the apoptosis inducing effect of this formula reported in a previous study.

**Methods:**

MCF-7 cells were treated with aqueous extracts of NDL at the IC_50_ concentration for 24, 36 and 48 h. Total RNAs at 24 h and 48 h were subsequently extracted, reverse transcribed and submitted to microarray expression profiling using the Human HT-12 v4.0 Expression Bead Chip (Illumina). Functional analyses were performed using the Database for Annotation, Visualization and Integrated Discovery and the Ingenuity Pathways Analysis. The expression level from selected genes at the three time points were assessed by quantitative real-time RT-PCR and Western blot.

**Results:**

Fifty-four and 601 genes were differentially expressed at 24 and 48 h of NDL treatment, respectively. Genes with altered expression at 24 h were mostly involved in cell responses to xenobiotic stress whereas genes differentially expressed at 48 h were related to endoplasmic reticulum stress, DNA damage and cell cycle control. Apoptosis of NDL treated MCF-7 cells resulted from a combination of different mechanisms including the intrinsic and extrinsic pathways, cell cycle arrest- and oxidative stress-related cell death.

**Conclusion:**

NDL elicited a two-stage response in MCF-7 treated cells with apoptosis as the ultimate result. The various mechanisms inducing apoptosis reflected the complexity of the formula composition.

**Electronic supplementary material:**

The online version of this article (10.1186/s12906-017-2027-2) contains supplementary material, which is available to authorized users.

## Background

Traditional medicine (TM) has been present in Vietnam for several thousand years, and was mostly based on the use of the local flora and fauna for disease prevention and treatment. Vietnamese traditional medicine, influenced by Traditional Chinese Medicine, employs a holistic and synergistic approach depending on the use of multi-ingredients formulae. Despite examples of successful treatment by TM, lack of scientific evidences, especially molecular mechanism of action, seriously hampered its development in the healthcare system [1]. Because of the holistic nature of TM, the identification of combined effects toward multiple molecular targets of multi-ingredients formulae poses a serious technological challenge. The microarray technology associated with data mining software constitutes a valuable tool for genome-wide analysis of molecular pathways underlying biological effects of TM [2]. Microarray-based transcriptional profiling was used in several studies to unravel the complex therapeutic effects of medicinal plants and traditional remedies [3, 4]. Molecular pathways activated or inhibited and genes modulated by TM treatment provide insights into its mechanisms of action.

Breast cancer is the leading cause of cancer mortality in women worldwide. It is the most frequently diagnosed malignancy in Vietnamese women over the last two decades [5]. Chemotherapeutic drugs for breast cancer varied depending on the cancer subtypes and other factors but have in common serious adverse effects on treated patients. TM, as an adjuvant therapy, may contribute to enhance the effectiveness of radiotherapy and chemotherapy while reducing the unwanted side effects. Some successful clinical trials for the treatment of breast cancer based on Traditional Chinese Medicine were reported, but the clinical effectiveness was not properly evaluated [6].

In a previous study, we founded that Nam Dia Long (NDL), a Vietnamese traditional formula empirically prescribed for the treatment of arthritis and some cancers, exhibited higher cytotoxicity on three cancer cell lines, MCF-7, Hep G2 and NCI-H460, compared to normal fibroblasts. This selective cytotoxicity was particularly important on MCF-7 cells, an estrogen-sensitive human breast cancer cell lines. Our results showed that NDL induced apoptosis on MCF-7 cells. Our results also indicated that synergistic interactions among four ingredients of the formula led to its overall biological activities [7]. In this study, we performed a microarray-based transcriptional profiling of MCF-7 cells treated with NDL for different periods of time. Selected genes identified from microarray data that were involved in cell responses leading to apoptosis were furtherly analyzed at the mRNA and protein levels.

The purpose of this study was to identify the possible mechanisms of action underlying the apoptosis inducing effects of NDL on MCF-7 cells.

## Methods

### Cell lines and cell culture

MCF-7 (HTB-22) cells were purchased from the American Type Culture Collection (ATCC, USA). Cells were cultured at 37°C and 5% CO_2_ in Eagle’s Minimal Essential Medium (EMEM) supplemented with 10% (*v*/v) FBS (Sigma, USA), 2 mM L-glutamine (Sigma, USA), 20 mM HEPES (Sigma, USA), 0.025 μg/mL amphotericin B (Sigma, USA), 100 IU/mL penicillin G (Sigma, USA), and 100 μg/mL streptomycin (Sigma, USA). Cells used in this study were between passages 4 and 20.

### Preparation of NDL extract

The NDL formula is composed of four ingredients: earthworm (*Pheretima aspergillum*), mung bean seed (*Vigna radiata* (L.) Wilczek), black bean seed (*Vigna unguiculata* (L.) Walp. subsp. unguiculata) and sweet leaf (*Sauropus androgynous* (L.) Merr.), all in the form of dried materials. These ingredients were identified and provided by the Traditional Medicine Hospital HCMC (Ho Chi Minh City, Vietnam). The quantity of NDL equivalent to one normal dosage for clinical use included 10 g earthworm, 20 g mung bean seed, 20 g black bean seed and 40 g sweet leaf in a final volume of 90 mL decoction. NDL extract was prepared as previously described [7]. To obtain a sufficient amount of material for all experiments performed in this study, a large quantity of NDL ingredients equal to many clinical doses was soaked in water for 20 min, boiled for 3 h in an automatic herbal extractor to obtain aqueous extract and lyophilized to obtain the dried powder. The extract yield of NDL was 0.08 g/g of dried material. Dried powders were stored at −80 °C. Before use, powders were dissolved in distilled water and 0.2 μm filter sterilized.

### RNA preparation

Cells at a density of 2 × 10^6^ cells in 10 cm-dish were incubated with NDL extracts at the IC_50_ concentration. After 24-, 36- and 48 h- incubation, total RNAs were extracted using RNeasy Mini Kit (Qiagen, Germany) according to the manufacturer’s protocol. RNA purity and integrity were assessed using a ND-1000 spectrophotometer (NanoDrop, USA) and Agilent 2100 Bioanalyzer (Agilent Technologies, USA). The RNA Integrity Number (RIN) was calculated for each sample, and RNA samples with RIN > 7.0 were considered for further analysis. The experiment was repeated at least three times.

### Microarray analysis

Microarray analysis was carried out by Macrogen (South Korea). Briefly, 500 ng of total RNA were amplified and purified using TargetAmp-Nano Labeling Kit for Illumina Expression BeadChip (Epicentre, USA) to yield biotinylated cRNA according to the manufacturer’s instructions. After that, 750 ng of labeled cRNA samples were hybridized to each Human HT-12 v4.0 Expression Beadchip (47,000 probes, Illumina, USA) for 18 h at 58 °C, according to the manufacturer’s instructions. The signal was detected using Amersham fluorolink streptavidin-Cy3 (GE Healthcare Bio-Sciences, UK) following the bead array manual. The quality of hybridization and overall chip performance were monitored by visual inspection of both internal quality control checks and the raw scanned data. Raw data were extracted using the software provided by the manufacturer (Illumina Genome Studio v2011.1 (Gene Expression Module v1.9.0)) and transformed by logarithm and normalized by quantile method. Local-pooled-error (LPE) test and fold change (fc) were used to identified significant differentially expressed genes. False discovery rate (FDR) was controlled by adjusting *p* value using Benjamini-Hochberg algorithm. Briefly, gene expression measured by probes with fc ≥ 2 & *p* < 0.05 & fail.count <6 probes was considered differential.

The Gene-Enrichment and Functional Annotation analysis for significant probe list was performed using DAVID Bioinformatics Resources 6.7 NIAID/NIH (http://david.abcc.ncifcrf.gov/home.jsp). The GO categories with *p* < 0.01, FDR < 0.05 and fold enrichment >1.5 were considered significant. The Ingenuity Pathways Analysis (IPA, Ingenuity Systems, http://www.ingenuity.com) was used to identify the canonical pathways and upstream regulators modulated by NDL treatment. Pathways with cut-off *p* ≤ 0.01 (identified by right-tailed Fisher’s exact test and Benjamini–Hochberg multiple testing correction) were considered for further analysis. A z-score value was used to assess the activation state of upstream regulators. A z-score value > 2 or < −2 defined a statistically significant activated or inhibited status, respectively.

### Quantitative real-time PCR (qRT-PCR)

Quantitative real-time RT-PCR was performed as previously described [8]. Briefly, total RNAs were isolated from control and NDL treated MCF-7 cells using Illustra RNAspin mini kit (GE Healthcare Life Sciences, UK) and reverse transcribed into complementary DNA by reverse transcription supermix (Agilent, USA) according to the manufacturer’s instruction. Real-time PCR was then performed in a volume of 40 μL containing gene-specific primers (Additional file 1: Table S1), 1 μg cDNA, EvaGreen and PCR master mix (SolGent, South Korea). PCR conditions were as follows: 95 °C for 15 min, 40 cycles of 95 °C at 30 s, 59 °C at 30 s, and 72 °C at 30 s. The relative expression was determined by the 2^-ΔΔCt^ method with GAPDH as internal control [9]. The statistical differences between the treated and control cells were determined by two-tail paired Student’s t-tests through the delta Ct values.

### Western blot analysis

Control and NDL treated MCF-7 cells were lysed by RIPA buffer (Thermo Scientific Pierce, USA) containing a protease inhibitor cocktail (Complete Protease Inhibitor Cocktail Tablets, Roche Diagnostics GmbH, USA). Protein supernatants were collected by centrifugation at 14,000 rpm for 15 min, 4°C. Protein concentration was measured by BCA Protein Assay kit (Thermo Scientific Pierce, USA). Equal amounts of cell lysate proteins were separated by SDS PAGE and transferred onto nitrocellulose membranes. After blocking by 5% skim milk in PBS (phosphate buffered saline), membranes were incubated overnight at 4 °C with primary antibodies (Santa Cruz, USA). Membranes were subsequently rinsed five times with 0.1% Tween in PBS, and incubated with horseradish peroxidase-conjugated secondary antibody (1: 5000) (Santa Cruz, USA) for 1 h at room temperature. Finally, membranes were washed five times with 0.1% Tween in PBS. Protein signals were visualized by SuperSignal West Pico Chemiluminescent Substrate (Thermo Scientific Pierce, USA) and scanned by ImageQuant LAS 500 (GE Healthcare Bio-Sciences, UK).

### Statistical analysis

Values were given as mean ± SD. Data were represented as averages of independentexperiments performed in triplicate and processed with GraphPad Prism 5 biostatistics software.

## Results

MCF-7 cells were treated with NDL at the IC_50_ concentration and collected at 24 h and 48 h after treatment for microarray analysis. Microarray data were processed using DAVID for Gene-Enrichment and Functional Annotation analysis, and the IPA for the identification of canonical pathways and upstream regulators. The validation of microarray data was performed by semi-quantitative PCR on five genes with varied profiles of differential expression. Our attention was furtherly focused on differentially expressed genes at 48 h which were closely involved in apoptosis induction of NDL treated MCF-7 cells. The expression of these genes was analyzed by qRT-PCR and Western blotting from MCF-7 cells treated with NDL for 24 h, 36 h, and 48 h.

### Identification of differentially expressed genes

From the microarray data, we assessed the treatment effects of NDL on MCF-7 cells by comparing treated to control cells. Applying cut-offs of *p* < 0.01 and fold change ≥ 2, genes with significant differential expression at 24 h and 48 h were identified. At 24 h, 44 and 10 genes were upregulated and downregulated, respectively. At 48 h, the number of genes upregulated and downregulated was 331 and 270, respectively (Additional file 2: Table S2). The top 10 differentially expressed genes at 24 h and 48 h treatment were showed in Table 1.

**Table 1:** Top 10 differentially expressed genes at 24 h and 48 h treatment

Upregulation				Downregulation			
24 h		48 h		24 h		48 h	
Gene	Fold change	Gene	Fold change	Gene	Fold change	Gene	Fold change
AKR1C4	11.81 ± 0.28	FAM129A	18.46 ± 0.25	TXNIP	−4.17 ± 0.65	TXNIP	−12.75 ± 0.43
AKR1C3	9.15 ± 0.18	GDF15	15.70 ± 0.27	GPER	−3.10 ± 0.25	AFAP1L2	−5.26 ± 0.32
AKR1C2	7.44 ± 0.21	DDIT3	14.73 ± 0.32	KCNK12	−2.79 ± 0.78	ID3	−4.52 ± 0.38
FGB	5.72 ± 0.73	FLJ35767	14.48 ± 0.22	AFAP1L2	−2.56 ± 0.34	EFEMP1	−4.27 ± 0.38
CYP1A1	5.30 ± 0.22	HSPA6	14.23 ± 0.49	SAMD11	−2.45 ± 0.24	NUDT1	−4.21 ± 0.18
GDF15	4.50 ± 0.20	LCN2	12.99 ± 0.27	KCNJ8	−2.39 ± 0.23	ID1	−4.05 ± 0.51
FGG	4.43 ± 0.48	AKR1C3	12.88 ± 0.31	EFEMP1	−2.29 ± 0.29	CCL2	−4.02 ± 0.38
ADM	4.18 ± 0.50	AKR1C4	11.33 ± 0.30	ID1	−2.12 ± 0.75	GPER	−3.99 ± 0.20
RASD1	4.15 ± 0.78	CYP1A1	11.27 ± 0.23	SMAD7	−2.02 ± 0.13	ELOVL2	−3.91 ± 0.19
ABCC3	4.11 ± 0.44	F7	10.84 ± 0.20	ID3	−2.00 ± 0.50	KCNJ8	−3.88 ± 0.19

Each value represents mean ± SD (*n* = 3)

The panel of genes upregulated at 24 h, such as AKR1C4, AKR1C3, AKR1C2, CYP1A1, was mainly involved in xenobiotic metabolism. The most up-regulated genes at 48 h included FAM129A, GDF-15, DDIT3, HSPA6, LCN2, AKR1C3, AKR1C4, CYP1A1, and F7. The most down-regulated gene at 24 h and 48 h was TXNIP. Other genes such as IL8, ANGPTL4, ADM were upregulated at 24 h followed by a downregulation at 48 h (Table 2). The majority of genes differentially expressed at 48 h also displayed altered expression at both time points (Table 2, Additional file 3: Table S3). These genes were mostly ER stress-related (FAM129A, GDF-15, DDIT3, HSPA6) and oxidative-related (LCN2, ANGPTL4, IL8, TXNIP), whose induction may regulate cell proliferation and apoptosis.

**Table 2:** Top differentially expressed genes at both time points

Symbol	Name	Fold change	
		24 h	48 h
Upregulated at 24 h and 48 h			
FAM129A	Family with sequence similarity 129 member A	1.80 ± 0.26	18.46 ± 0.25
GDF15	Growth differentiation factor 15	4.50 ± 0.20	15.70 ± 0.27
DDIT3	DNA damage inducible transcript 3	2.39 ± 0.66	14.73 ± 0.32
FLJ35767		2.30 ± 0.42	14.48 ± 0.22
HSPA6	Heat shock protein family A (Hsp70) member 6	3.49 ± 0.28	14.23 ± 0.49
LCN2	Lipocalin 2	1.54 ± 0.40	12.99 ± 0.27
AKR1C3	Aldo-keto reductase family 1 member C3	9.15 ± 0.18	12.88 ± 0.31
AKR1C4	Aldo-keto reductase family 1 member C4	11.81 ± 0.28	11.33 ± 0.30
CYP1A1	Cytochrome P450 family 1 subfamily A member 1	5.30 ± 0.22	11.27 ± 0.23
F7	Coagulation factor VII	1.65 ± 0.10	10.84 ± 0.20
Down-regulated at 24 h and 48 h			
TXNIP	Thioredoxin-interacting protein	−4.17 ± 0.65	−12.75 ± 0.43
AFAP1L2	Actin filament associated protein 1 like 2	−2.56 ± 0.34	−5.26 ± 0.32
ID3	Inhibitor of DNA binding 3	−2.00 ± 0.50	−4.52 ± 0.38
EFEMP1	EGF containing fibulin like extracellular matrix protein 1	−2.29 ± 0.29	−4.27 ± 0.38
NUDT1	Nudix hydrolase 1	−1.52 ± 0.08	−4.21 ± 0.18
ID1	Inhibitor of DNA binding 1	−2.12 ± 0.75	−4.05 ± 0.51
KCNJ8	Potassium voltage-gated channel subfamily J member 8	−2.39 ± 0.23	−3.88 ± 0.19
GPER	The G-protein-coupled estrogen receptor-1	−3.10 ± 0.25	−3.41 ± 0.17
ID2	Inhibitor of DNA binding 2	−1.84 ± 0.23	−3.13 ± 0.20
Up-regulated at 24 h, down-regulated at 48 h			
ANGPTL4	Angiopoietin-like 4	2.74 ± 0.40	−2.72 ± 0.35
IL8	Interleukin-8	2.72 ± 0.61	−2.56 ± 0.36
ADM	Adrenomedullin	4.18 ± 0.50	−1.84 ± 0.61

Each value represents mean ± SD (*n* = 3)

Fam129A (NIBAN) is upregulated in response to ER stress and can modulate cell death signaling through positively regulating protein synthesis [10]. The growth differentiation factor 15 (GDF15), a member of the transforming growth factor β superfamily, is induced by diverse cellular stress signals. GDF15 can have opposite effects, antitumorigenic or tumorigenic, depending on cell types, disease stage or microenvironment. As an antitumoral factor, GFD15 is an important downstream target of p53, inhibits cell growth and induces apoptosis in various tumor cells including MCF-7 cells [11]. An upregulation of NIBAN and GDF15 expression at 24 h that was furtherly increased at 48 h suggested an important increasing pression of ER stress in NDL treated MCF-7 cells. DDIT3 is a transcription factor with a majority of target genes involved in cell migration, cell cycle control and apoptosis induction [12]. DDIT3 is upregulated in response to various stress conditions, and is a key mediator of ER-induced cell death. The overexpression of DDIT3 and the decreased expression of BCL2, its downstream target, observed in this study were probably responsible of antiproliferative and apoptotic effects of NDL on MCF-7 cells. LCN2 is a multi-faceted protein with contrasting effects on tumorigenesis and metastasis depending on species and cell lineages specific responses [13]. LCN2 activities were attributed to its iron scavenging properties that can attenuate iron-related oxidative stress [14]. The coagulation factor VII (F7) participates in maintaining vascular hemostasis and promotes breast cancer cell proliferation, invasion and metastasis [15]. The overexpression of LCN2 and F7 observed in this study suggested a protective effect of NDL on MCF-7 cells. Among the downregulated genes in this study, thioredoxin-interacting protein (TXNIP) had the most decreased expression with a fold change > 3. TXNIP is induced by ER stress and regulates the activity of protein disulfide isomerases (PDIs). It binds to PDIs and increases PDIs activity thus is a feedback regulator of UPR signaling to decrease ER stress [16]. ID1 and ID3 promote angiogenesis during tumor growth. Overexpression of ID1 facilitates the G1 to S phase transition, and induces breast cancer metastasis [17]. NUDT1 is involved in oxidative stress protective mechanism necessary for tumor cells survival since tumors produce large amounts of oxidants [18]. The decreased expression of ID1, ID3, NUDT1 observed in this study had antiproliferative and pro-apoptotic activities whereas TXNIP downregulation could result in protective effect on NDL treated MCF-7 cells. In this study, several genes such as ANGPTL4, IL8, ADM, were upregulated at 24 h and downregulated at 48 h. ANGPTL4 is a tumor suppressor that inhibits angiogenesis [19]. The inflammatory cytokine IL8 is reported to participate in proliferation, angiogenesis, invasion and metastases during cancer progression [20]. ADM plays important role in apoptosis inhibition, immune escape, and angiogenesis [21]. Up- and downregulated genes induced by NDL on MCF-7 cells at 48 h displayed opposing activities with a dominance of cell death inducing effect.

In brief, the expression profile of differentially expressed genes at 24 h and 48 h suggested an early response for survival followed by apoptotic cell death in NDL treated MCF-7 cells.

### Analysis of canonical pathways by IPA

The GO term analysis was performed for functional classification of the differentially expressed genes between control and NDL treated MCF-7 cells by DAVID. The GO enrichment analysis showed that the common gene set at 24 h was significantly enriched in cellular responses to xenobiotic stresses. At 48 h, the gene set was significantly enriched in cell cycle-related processes (Additional file 4: Table S4), with few genes involved in protein-related stress.

**Fig. 1:**
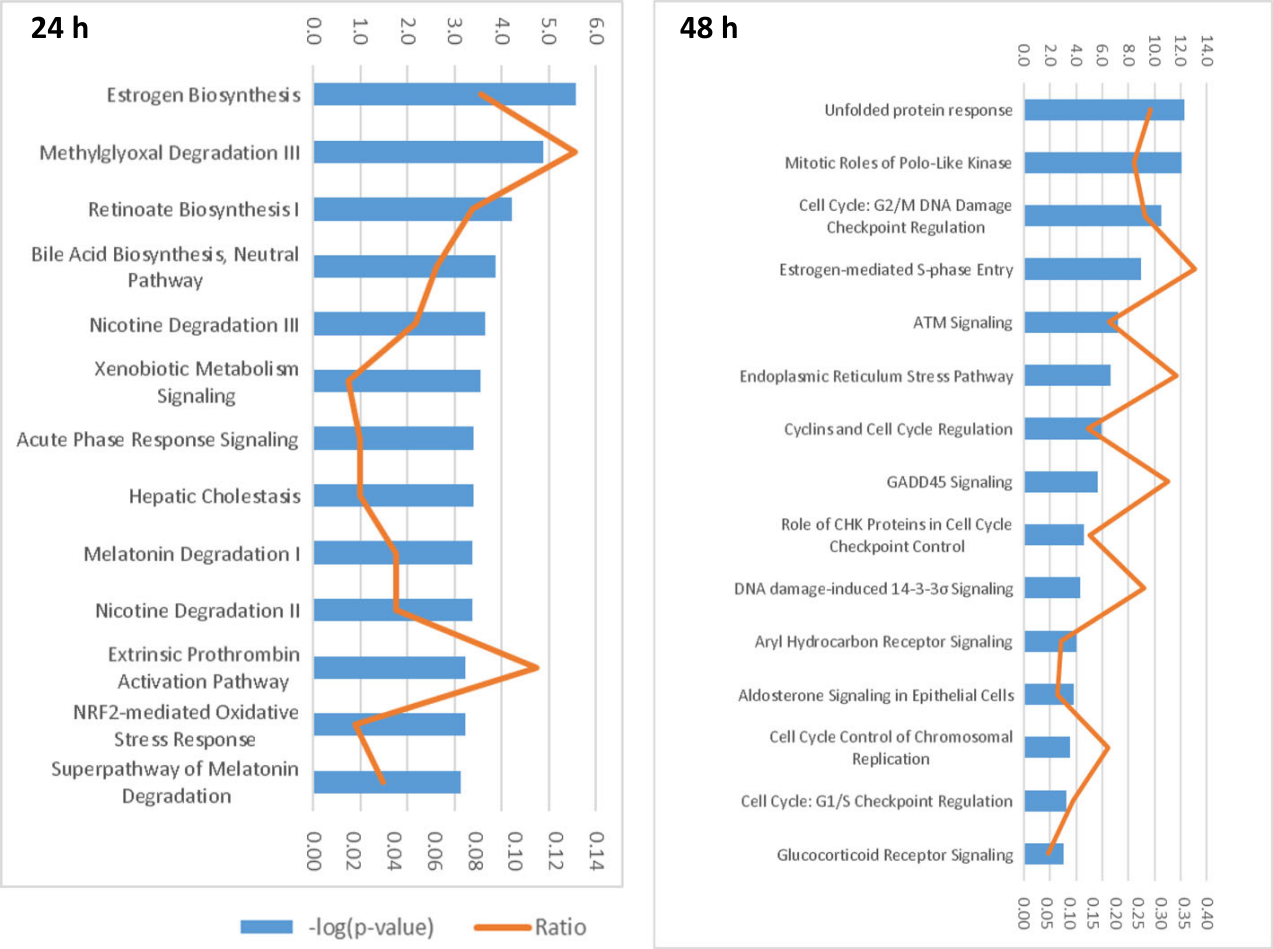
Top IPA canonical pathways identified at 24 h and 48 h after NDL treatment of MCF-7 cells (*p* ≤ 0.001)

We subsequently used the IPA software to predict canonical pathways underlying NDL treatment effects on MCF-7 cells. Based on the same cut-off of *p* ≤ 0.01, the IPA identified 27 and 30 canonical pathways at 24 h and 48 h, respectively. These pathways were ranked according to the *p* value in a descending order (Additional file 5: Table S5). The top pathways at 24 h with *p* ≤ 0.001 included those involved in early phase responses to endogenous and exogenous stresses whereas at 48 h they were mostly related to cell cycle, unfolded protein response and DNA damage (Fig. 1). Interestingly, Estrogen Biosynthesis, Methylglioxal Degradation III, Retinoate Biosynthesis I, the top 3 pathways at 24 h were found among the last ranked pathways at 48 h. Contrarily, Unfolded Protein Response, Endoplamic Reticulum Stress Pathway and Aryl Carbon Receptor Signaling which were at the last positions at 24 h were ranked at or near the top at 48 h (Additional file 5: Table S5). However, pathways at 24 h were represented by small numbers of genes, and even with a p cut-off ≤0.001, should be interpreted with caution. Some pathways specifically involved in breast cancer were found among canonical pathways predicted by the IPA at 24 h and 48 h, included Estrogen-Mediated S Phase Entry, Hereditary Breast Cancer Signaling, Breast Cancer Regulation by Stathmin 1, Estrogen Synthesis (Fig. 1 and Additional file 5).

### Upstream regulators (URs) predicted by the IPA

Using cut-offs of *p* ≤ 0.001 and z-score ≥ 2 and ≤ −2, we obtained a list of 49 URs at 24 h and 196 URs at 48 h (Additional file 6: Table S6). The top predicted URs at 24 h and 48 h were displayed in Table 3. Doxorubicin, an anthracycline used in breast cancer treatment was predicted for both time points. The key regulator of ER stress TP53 was listed as the first UR at 48 h, followed by CDKN1A, the cyclin-dependent kinase inhibitor. The most inhibited URs at 48 h predicted by the IPA were RABL6 and ERBB2. RABL6 belongs to the RAB proteins family, considered as the key regulators of intracellular trafficking that can promote proliferation, migration and invasion of tumor cells through co-ordinating different signaling pathways cross-talk [22]. ERBB2 is a member of the epidermal growth factor family of transmembrane receptors whose overexpression drives oncogenic cell proliferation [23]. Interestingly, thapsigargin, an inhibitor of the sarco/endoplasmic reticulum Ca2+ ATPase (SERCA), was listed as an UR at 24 h (p 1.5E-05, z-score 2.2) and at 48 h (p 4.4E-10, z-score 2.9) (Additional file 6). In our previous work, cell growth real-time monitoring of NDL treated MCF-7 cells suggested a mechanism similar to that exerted by thapsigargin [7].

**Table 3:** Top URs predicted by the IPA at 24 h and 48 h after NDL treatment of MCF-7 cells

Upstream Regulator	Molecule Type	*p* value	z-score	Upstream Regulator	Molecule Type	*p*-value	z-score
24 h				48 h			
Activated							
Doxorubicin	chemical drug	1.2E-16	3.32	TP53	transcription regulator	9.8E-50	5.38
Cigarette smoke	chemical toxicant	2.8E-15	2.07	CDKN1A	kinase	1.3E-48	4.11
TNF	cytokine	1.6E-13	2.27	NUPR1	transcription regulator	1.6E-32	6.43
Hydrogen peroxide	chemical - endogenous mammalian	1.9E-11	2.07	Calcitriol	chemical drug	1.6E-30	4.73
NFE2L2	transcription regulator	1.0E-10	2.68	Doxorubicin	chemical drug	6.1E-23	3.33
Inhibited							
SFTPA1	transporter	4.5E-09	−2.45	RABL6	other	1.9E-38	−5.40
Actinomycin D	chemical drug	4.6E-09	−2.13	ERBB2	kinase	1.0E-29	−2.37
N-acetyl-L-cysteine	chemical drug	1.8E-06	−2.36	E2F1	transcription regulator	5.4E-28	−3.41
SB203580	chemical - kinase inhibitor	3.5E-06	−2.37	Beta-estradiol	chemical - endogenous mammalian	7.7E-28	−2.02
				CSF2	cytokine	2.0E-27	−5.58

### Validation of microarray data by real-time RT-PCR

To assess the reliability of microarray data, we performed qRT-PCR on five differentially expressed genes including ERN1, GADD45A, BCL2, HMOX1 and ESR1. These genes displayed varied profiles of differential expression, up- and downregulation for both time durations or down- followed by upregulation. PCR results showed good correlation with microarray data with some differences in the magnitude of fold change (Table 4).

**Table 4:** Fold changes in expression of 5 genes obtained from qRT-PCR and microarray data

Gene	Microarray			qRT-PCR		
	24 h	48 h	24 h/48 h ratio	24 h	48 h	24 h/48 h ratio
ERN1	1.19 ± 0.10	2.26 ± 0.23	1.9	1.68 ± 0.32	2.10 ± 0.53	1.25
GADD45A	1.60 ± 0.10	3.84 ± 0.07	2.4	2.84 ± 1.17	6.15 ± 3.43	2.2
CDKN1A	−1.22 ± 0.22	2.35 ± 0.12	2	−0.95 ± 1.98	2.91 ± 0.69	3
HMOX1	3.97 ± 0.17	10.05 ± 0.30	2.5	5.69 ± 0.90	17.40 ± 7.54	3
ESR1	−1.33 ± 0.12	−2.14 ± 0.11	1.6	−1.23 ± 0.09	−1.66 ± 0.87	1.3

Each value represents mean ± SD (*n* = 3)

### Effects of NDL on MCF-7 cells gene expression analyzed by real-time RT-PCR and western blot

**Fig. 2:**
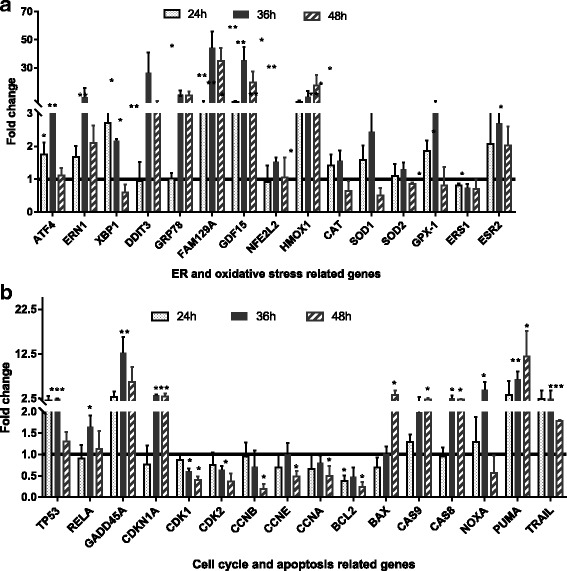
Real-time RT-PCR analysis of gene expression level after treating MCF-7 cells with NDL for 24, 36 and 48 h. Fold change indicated relative quantitation normalized to GADPH. Fold change >1 and <1 indicated upregulation and downregulation, respectively. The statistical differences between the treatment and control conditions were analyzed by two-tailed paired Student’s t-tests (*p < 0.05; **p < 0.01; ***p < 0.001). **a**, ER and oxidative stress related genes; **b**, cell cyle and apoptosis related genes

**Fig. 3:**
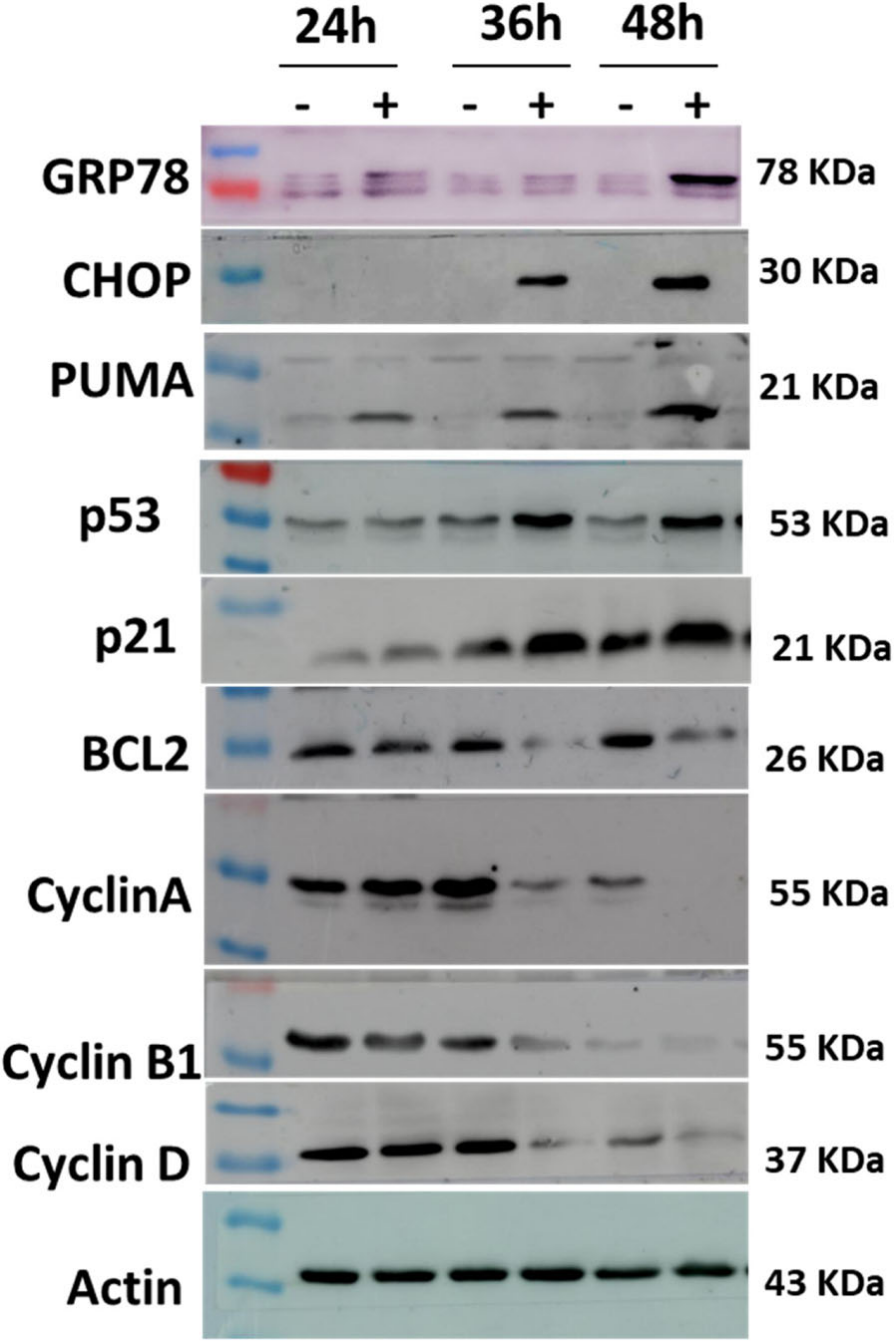
Western blot analysis of some differentially expressed genes in NDL treated MCF-7 cells

We subsequently analyzed 31 genes involved in ER and oxidative stresses, apoptosis, and cell cycle control at 24 h, 36 h and 48 h at the mRNA and protein levels (Fig. 2 and Fig. 3). MCF-7 cells were treated with NDL at the IC_50_ dose for 24, 36 and 48 h. NDL was replaced by distilled water for the control cells. Total RNAs from control and treated cells were extracted and reverse transcribed. Real-time PCR was performed with gene-specific primers. The 2^-ΔΔCt^ method was used to assess the relative change in gene expression normalized to GADPH internal control. A t-test *p* < 0.05 was considered as significant. Results shown in Fig. 2 and Table 5 were in accordance with those obtained through transcriptional profiling. Furthermore, since an intermediate time point (36 h) was added for analysis, the kinetic of gene expression alteration of some genes was more precisely defined.

**Table 5:** Real-time RT-PCR analysis of gene expression level after treating MCF-7 cells with NDL for 24, 36 and 48 h

Gene	Realtime RT-PCR		
	24 h	36 h	48 h
ER stress			
GRP78	1.02 ± 0.16	10.82 ± 3.05**	10.06 ± 3.05**
ERN1	1.68 ± 0.32	8.86 ± 6.54*	2.10 ± 0.53*
ATF4	1.75 ± 0.37*	3.67 ± 0.61**	1.11 ± 0.24
XBP1	2.71 ± 1.84	2.17 ± 0.06**	0.60 ± 0.24
DDIT3	0.94 ± 0.59	26.45 ± 14.47*	4.56 ± 1.57*
FAM129A	4.52 ± 1.84*	44.20 ± 11.70**	34.84 ± 9.14**
GDF15	5.65 ± 0.72**	35.36 ± 9.23*	19.49 ± 7.78**
p53 pathway			
TP53	2.06 ± 0.95	2.46 ± 0.05***	1.30 ± 0.23
RELA	0.90 ± 0.32	1.65 ± 0.26*	1.14 ± 0.41
GADD45A	2.84 ± 1.17	12.82 ± 3.45**	6.15 ± 3.43*
CDKN1A	0.76 ± 0.46	3.26 ± 0.17***	2.91 ± 0.69*
Cell cycle			
CDK1	0.88 ± 0.12	0.61 ± 0.07*	0.41 ± 0.08*
CDK2	0.75 ± 0.29	0.64 ± 0.08*	0.37 ± 0.18
CCNB	0.95 ± 0.33	0.71 ± 0.38	0.19 ± 0.12*
CCNE	0.70 ± 0.32	1.01 ± 0.26	0.48 ± 0.13*
CCNA2	0.66 ± 0.33	0.81 ± 0.17	0.50 ± 0.24*
Apoptosis			
BCL2	0.37 ± 0.12*	0.48 ± 0.21	0.25 ± 0.11*
BAX	0.70 ± 0.23	1.02 ± 0.17	3.28 ± 1.03*
CAS9	1.30 ± 0.17	1.99 ± 0.87	2.23 ± 0.44*
CAS8	0.96 ± 0.21	2.52 ± 0.62*	2.30 ± 0.14*
NOXA	1.29 ± 0.59	4.43 ± 1.75*	0.57 ± 0.40
PUMA	3.31 ± 3.10	6.76 ± 1.75**	11.95 ± 5.74*
TRAIL	2.44 ± 1.87	2.44 ± 1.82	1.78 ± 0.03***
Oxidative stress response			
NFE2L2	0.91 ± 0.51	1.53 ± 0.13*	1.05 ± 0.61
HMOX1	5.69 ± 0.90**	9.37 ± 3.96*	17.40 ± 7.54*
CAT	1.41 ± 0.33	1.56 ± 0.31	0.64 ± 0.31
SOD1	1.59 ± 0.43	2.44 ± 0.95	0.50 ± 0.23
SOD2	1.10 ± 0.37	1.30 ± 0.21	0.85 ± 0.05*
GPX-1	1.87 ± 0.31*	3.83 ± 2.50	0.80 ± 0.57
Estrogen receptor			
ESR1	0.81 ± 0.06*	0.73 ± 0.11	0.70 ± 0.29
ESR2	2.08 ± 1.20	2.70 ± 0.61*	2.03 ± 0.58

Each value represents mean ± SD (*n* = 3). Fold change indicated relative quantitation compared to GADPH. Fold change >1 indicates upregulation while fold change < 1 indicates down regulation. The statistical differences between the treatment and control were analyzed by two-tailed paired Student’s t-tests (*p < 0.05; **p < 0.01; ***p < 0.001)

In NDL treated MCF-7 cells, all three arms of the unfolded protein response (UPR) including ERN1, ATF6 and PERK downstream target ATF4 were overexpressed at the three time points, with a sharp increase at 36 h (Fig. 2). Key ER stress markers such as GRP78, DDIT3 were markedly upregulated at 36 h and 48 h (Fig. 2 and Fig. 3). An upregulation of FAM129A at the three time points and a sharp increase of GDF15 expression at 36 h and 48 h, in accordance with microarray data, indicated an increasing pression of ER stress. Oxidative stress, though less pronounced, was revealed through NFE2L2 upregulation, and particularly through the highly overexpression of HMOX1 gene. NFE2L2, the inducer of many antioxidant proteins, plays important role in driving the expression of antioxidant genes such as FTH1, FTL, HMOX1, AKR, GST [24]. In MCF-7 treated cells, it was slightly up-regulated at 36 h whereas heme oxygenase-1 (HMOX1), an endoplasmic reticulum resident protein and NFE2L2 target gene, was increasingly upregulated to reach a 17-fold change compared to untreated conditions. HMOX1, highly induced under oxidative stress, was shown to exert antiproliferative and proapoptotic effects on some rat and human breast cancer cell lines. Its overexpression also inhibits the invasiveness and migration of breast cancer cells through suppression of matrix metalloproteinase 9 expression [25]. However, the expression of other antioxidative enzyme encoding genes such as CAT, SOD1, SOD2 and GPX-1 decreased at 48 h of NDL treatment.

The tumor suppressor gene TP53 induces the cyclin-dependent kinase inhibitor CDKN1A expression leading to an inhibition of cyclin D/CDKs, thus causing a G1 arrest. It can also induce a G2 arrest cell type-specific, and inhibits entry into S phase when the mitotic spindle has been damaged [26]. In p53-dependent gene repression, p21 downregulates genes responsible for cell cycle progression such as CDC25C, CDC2, CHEK1, CCNB1 among others [27]. In this study, the enhanced expression of TP53 and CDKN1A together with progressively down-regulation of genes involved in cell cycle control such as CDK1, CDK2, CCNB, CCNE, CCNA2 from 24 h to 48 h suggested an antiproliferative effect of NDL through non-phase specific cell cycle arrest (Fig. 2b).

In NDL treated MCF-7 cells, several apoptosis-related genes displayed significant differential expression including upregulated PUMA, NOXA, TP53, CDKN1A, GADD45A, CAS9, BAX, TRAIL and downregulated BCL2. PUMA, a BCL2 homology 3 (BH3)-only Bcl-2 family member, is transactivated by p53 in response to diverse stimuli including genotoxic stresses such as DNA damage, toxins, proteasome and transcription inhibitors. PUMA promotes mitochondrial outer membrane permeabilization leading to cell death by cooperating with BCL2 effectors BAX and BAK [28]. NOXA, another (BH3)-only protein is a primary p53-response gene. It enhances the oligomerization of BAX or BAK leading to apoptosis induction after DNA damage. The apoptosis inducing effect of NOXA may be more relevant in highly proliferating or malignant versus differentiated, normal cells [29]. After DNA damage, GADD45A family members are upregulated, resulting in cell cycle arrest and apoptosis. GADD45A exerts cell cycle arrest effect by targeting the CDK1/Cyclin B1 complex that is responsible for the G2/M checkpoint. Furthermore, GADD45A proteins can interact with p21 to cause cell cycle arrest at both G1/S and G2/M transitions [30]. BCL2 family proteins control cell survival through preserving the integrity of mitochondrial outer membrane. Pro-apoptotic BCL2 effectors such as BAX and BAK are activated under sustainable cellular stress. In the extrinsic pathway of apoptosis, TRAIL selectively induces apoptosis in various tumor cells over normal cells through interacting with its receptors leading to the activation of caspase 8 and 10 [31]. The overexpression of TRAIL, and an upregulation though not significant of TRAIL-R4 and caspase 8 in this study also suggested a mechanism of extrinsic pathway of apoptosis. Furthermore, estrogen receptor alpha was downregulated whereas estrogen receptor beta was upregulated. In estrogen sensitive tissues such as breast, occupancy of ERα is considered as pro-proliferative whereas occupancy of ERβ is considered as antiproliferative [32]. In brief, the killing effect of NDL on MCF-7 cells resulted from excessive ER stress combined to oxidative stress, cell cycle arrest and apoptosis induction through the intrinsic and the extrinsic pathways.

Western blot analysis of GRP78, CHOP (DDIT3), PUMA, p53, p21, BCL2, cyclin A, cyclin B1 and cyclin D in NDL treated MCF-7 cells at 24 h, 36 h and 48 h were consistent with qRT-PCR data (Fig. 3). Key ER stress markers GRP78, CHOP were markedly and progressively upregulated at the three time points. The antiapoptotic protein p53 and its downstream regulators PUMA and p21 were overexpressed whereas anti-apoptotic protein BCL2 was underexpressed. The concentration of cyclin A, cyclin D and cyclin B1 decreased progressively from 24 h to 48 h.

## Discussion

In our previous study, we showed that NDL treatment induced apoptosis on several tumor cell lines, especially MCF-7 cells. The apoptotic inducing effect of NDL resulted from synergistic interactions among different components of the formula and was not cell cycle phase specific [7]. To investigate the molecular mechanisms underlying apoptosis inducing effects of NDL on MCF-7 cells, we profiled a global mRNA expression analysis. Using causal analytics approaches implemented in the IPA, we identified genes and signaling pathways differentially expressed by NDL treated MCF-7 cells. Real-time RT-PCR and Western blot were used to further analyzing important pathways involved in cell death.

### NDL elicited a two-phase response in MCF-7 cells

We analyzed the effects of NDL treatment for different durations of time, 24 h, 36 h and 48 h. Exposure to plant extracts for 24 h is sufficient to elicit cell responses at the gene expression level [33]. Longer exposure times allowed analysis of the transcriptional cascade that led to the beginning of cell death at 48 h after NDL treatment [7].

We observed a two-phase response of treated cells, an early response to xenobiotic stresses at 24 h and an apoptosis process at 48 h. The top most upregulated genes at 24 h revealed by microarray analysis including AKR1C4, AKR1C3, AKR1C2, CYP1A1 were involved in xenobiotic metabolism. At 48 h, ER stress- and oxidative-related genes were predominant. The increased expression at mRNA and protein levels of PUMA, NOXA, DDIT3, TRAIL, CAS9, BAX at 36 h and 48 h clearly indicated an induction of apoptosis leading to cell death as previously reported [7]. The IPA predicted canonical pathways confirmed these observations. The Methylglyoxal Degradation III, Nicotine Degradation III and II, Xenobiotic Metabolism Signaling identified among the top 10 pathways at 24 h indicated an effort for xenobiotics elimination essential for cell survival. At 48 h we observed a predominance of pathways related to ER stress, UPR, DNA damage and cell cycle control; all of them can ultimately lead to apoptosis. Cell cycle: G2/M DNA Damage Checkpoint Regulation, ATM Signaling, GADD45 Signaling, DNA Damage-Induced 14–3-3σ Signaling found among the top 10 pathways identified at 48 h strongly suggested a major role of DNA damage causing cell death. The ranking position switch between 24 h- (Estrogen Biosynthesis, Methylglioxal Degradation III, Retinoate Biosynthesis I) and 48 h- (Unfolded Protein Response, Endoplamic Reticulum Stress Pathway and Aryl Carbon Receptor Signaling) predicted pathways also indicated an early survival response of treated cells which was further replaced by apoptosis induction. Different cell cycle phase related pathways identified at 48 h such as Mitotic Roles of Polo-Like Kinases, Cell Cycle:G2/M DNA Damage Checkpoint Regulation (activated), Cyclins and Cell Cycle Regulation, Role of CHK Proteins in Cell Cycle Regulation, Cell Cycle: G1/S Checkpoint Regulation (activated) indicated a non-phase specific arrest effect of NDL on MCF-7 cells as previously reported [7]. Interestingly, Estrogene Biosynthesis, the first ranked pathway at 24 h, exhibited less importance at 48 h whereas Estrogen-Mediated S-Phase Entry (ranked at position 4) was clearly inhibited at 48 h (Additional file 5: Table S5).

### The apoptosis inducing effect of NDL resulted from a combination of mechanisms of action

The profile of differentially expressed genes at 24 h and 48 h revealed different mechanisms of action of NDL on MCF-7 cells, including ER stress, oxidative stress, intrinsic and extrinsic pathways of apoptosis. The ER stress response promotes cell survival by reducing protein synthesis and upregulating chaperones. However, if the UPR persists and ER stress can not be resolved, cells will undergo apoptotic process [34]. ER stress followed by UPR activation were probably the main causes of NDL treated MCF-7 cells apoptosis at 48 h, indicated by differentially expressed key factors including ATF4, CHOP, PUMA, BCL2, ERN1. Apoptosis of treated MCF-7 cells was triggered mainly through the mitochondrial intrinsic pathway denoted by the overexpression of numerous key factors such as PUMA, NOXA, BAX, CAS9, BCL2. Besides, an increase of TRAIL and caspase 8 expression at 48 h suggested the contribution of the extrinsic apoptosis pathway as previously described [35]. The upregulation of NFE2L2 at 36 h and HMOX1 at the three time points observed in this study also indicated the presence of oxidative stress response. Glucocorticoid Receptor signaling and NRF2-Mediated Oxidative Stress Response, Extrinsic Prothrombin Activation Pathway and Airway Pathology in Chronic Obstructive Pulmonary Disease present at both timing in NDL treated MCF-7 cells confirmed a ubiquitous though less pronounced role of oxidative stress. The crosstalk between ER stress and oxidative stress can be mediated by DDIT3, an UPR component which can induce oxidative stress [36]. The upregulation of DDIT3 in this study could play a role in the co-ordination of ER and oxidative stresses in NDL treated cells. The numerous URs identified for 24 h and 48 h of NDL treatment highlighted the complex pharmacological effects of NDL on MCF-7 cells. Their mechanisms of action differed and can complement each other. Interestingly, in our previous work, kinetic profile of NDL treated MCF-7 cells growth suggested an effect similar to that of thapsigargin, an inhibitor of the SERCA [7]. IPA analysis in this study predicted thapsigargin as an UR at both time durations with *p* values of 1.5E-05, 4.4E-10, respectively and an activation z-score of 2.2 and 2.9, respectively. The inhibition of SERCA by thapsigargin affects the calcium homeostasis and can activate apoptotic pathways causing cell death [37]. NDL selectively induced cell death in MCF-7 cells but not in normal cells as previously shown in our previous work. In this study, we showed that NDL activates the UPR, converting it from cytoprotective to cytotoxic. A possible explanation for the selective cytotoxicity of NDL is that the UPR is already elevated in tumor cells, whereas it is of much lower level in normal cells. Therefore, it is much easier to increase the UPR to a level of conversion in tumor cells as suggested by previous study [38]. The complex pharmacological nature of NDL was also displayed through its effects on genes of opposing activities. CYP1A1, CYP2F1, CYP1B1 were upregulated while TXNIP was downregulated, though all of them were involved in ROS production. An enhanced expression of the antioxidant enzyme HO-1 and down-regulated CAT, SOD 1,2 and GPX were also revealed. These contradictory results probably reflected complicated interactions among NDL ingredients, either synergistic or antagonistic. Nevertheless, NDL seemed to induce a two-phase response in MCF-7 cells, a survival phase and an apoptotic phase, depending on exposure times.

### Anticancer activities of NDL on MCF-7 cells mimicked the mechanisms of action of some anticancer drugs for breast cancer

The most similar anticancer drugs to NDL among the top URs predicted were doxorubicin for 24 h; and calcitriol, doxorubicin, fulvestrant, medroxyprogesterone acetate for 48 h (Additional file 6: Table S6). Among these drugs, doxorubicin, fulvestrant, medroxyprogesterone acetate were FDA-approved drugs for breast cancer treatment.

Calcitriol acting as a steroid hormone regulates the expression of more than 60 target genes including p21, cyclins and CDKs. The upregulation of p21 by calcitriol accompanied by the reduced expression of cyclins A2, B1, B2, D, D3, E1, F, CDK2 and CDK4 blocks the transition from G1 to S phase and initiates the differentiation process. In ERα-negative breast cancer cells, calcitriol restores the response to antiestrogen such as tamoxifen and fulvestrant. It may work synergistically with other antineoplastic drugs such as taxanes, alkylating agents, and ionizing radiation [39]. Results from this study showed an increased expression of p21 and decreased expression of some cyclins and cyclin-dependent kinases at the mRNA and protein levels suggesting a mechanism of action similar to calcitriol on MCF-7 cells. Doxorubicin can act in cancer cells through two mechanisms: (1) intercalation into DNA and disruption of topoisomerase II-mediated DNA repair; and (2) free radical generation causing damage to cell membranes and DNA. The first pathway may be modulated by genes involved in the deactivation of free radicals such as glutathione peroxidase (GPX), superoxide dismutase (SOD), catalase while the second mechanism involves genes of the DNA repair system such as p53, TOP2A, and GADD45A. Furthermore, doxorubicin is a p53-dependent downregulator of stathmin (STMN1) [40]. Since high levels of stathmin may confer resistance to antimicrotubule agents [41]; a decreased expression would strengthen therapeutic effects of drugs such as vinblastine. In this study, we observed a decreased expression of CAT (fc −1.23), SOD1 (fc −2.0), SOD2 (fc −1.47), GPX1 (fc −1.25), TOP2A (fc −2.46) and stathmin at 48 h (fc −2.44). Fulvestrant, an estrogen receptor antagonist, competitively binds to the estrogen receptor and prevents endogenous estrogen effects on target cells. The combination of antiestrogens with antitumor agents has been shown to be more effective at inhibiting breast tumor cell lines than either drugs alone [42].

This study had some limitations. The first, which is common to other transcriptional profiling studies, was the lack of certainty about functional relationships among molecules and pathways revealed through microarray data mining of MCF-7 cells treated with NDL. However, the functional assays performed on selected genes strongly supported the prediction obtained from genome-wide analysis. Another problem involved the lack of a large panel of doses and durations of treatment, especially when dealing with a mixture of extracts that can have synergistic as well as antagonistic interactions depending on the dose and exposure time. Since this study was focused on apoptosis induction effects of NDL, other activities of the formula susceptible for use as an adjuvant therapy, which constituted the strength of traditional medicine, were not investigated. Moreover, the ideal level of UPR signaling to create apoptosis inducing effect instead of an adaptive survival UPR was not determined. Another limitation of this work, inherent to all in vitro studies, involved problems in extrapolating in vitro data to in vivo studies. Drug bioavailability, distribution, metabolism and clearance in in vivo systems as well as the complex interactions with different cell types were not truly reflected in in vitro experiments. However, results in this study did reveal the principal molecular pathways which constituted the targets for future in vivo studies [43].

## Conclusion

Traditional medicine based on the combination of herbs targeting different molecules/pathways may increase therapeutic efficacy and reduces adverse effects. However, traditional formulae probably contain also non-effective, even undesired ingredients or non-appropriate concentration of ingredients. The results of this study showed that NDL formula induced apoptosis on MCF-7 cells through different mechanisms, ER stress being the most important cause of cell death. But results also suggested counteracting actions among the ingredients of NDL formula. Since our previous work showed that the presence of all ingredients is needed for the highest cytotoxic effect, further investigations will be necessary to determine the dosage of each component to create the optimal apoptosis induction effect and microenvironment homeostasis susceptible for use as complementary medicine with conventional therapies.

## Additional files

Additional file 1: Table S1. Primers used for Real-time RT-PCR. (XLSX 12 kb)

Additional file 2: Table S2. Differentially expressed genes in MCF-7 cells after treatment with NDL at 24 h and 48 h. (XLSX 45 kb)

Additional file 3: Table S3. Genes differentially expressed at both 24 h and 48 h of treatment with NDL. (XLSX 15 kb)

Additional file 4: Table S4. The GO term analysis by DAVID of genes differentially expressed by MCF-7 cells treated with NDL. (XLSX 18 kb)

Additional file 5: Table S5. IPA canonical pathways identified at 24 h and 48 h from NDL treated MCF-7 cells. (XLSX 17 kb)

Additional file 6: Table S6. Upstream regulators predicted by the IPA at 24 h and 48 h after NDL treatment of MCF-7 cells. (XLSX 36 kb)

## Abbreviations

DAVID: Database for Annotation, Visualization and Integrated Discovery
ER: endoplasmic reticulum
Fc: fold change
FDR: False discovery rate
IPA: Ingenuity Pathways Analysis
NDL: Nam Dia Long
SERCA: sarco/endoplasmic reticulum Ca2+ ATPase
TM: traditional medicine
UPR: unfolded protein response
UR: upstream regulator
